# Fibrosis-4 index at diagnosis is associated with all-cause mortality in patients with microscopic polyangiitis and granulomatosis with polyangiitis

**DOI:** 10.1186/s12876-019-1007-z

**Published:** 2019-06-13

**Authors:** Hee Jin Park, Jun Yong Park, Seung Min Jung, Jason Jungsik Song, Yong-Beom Park, Sang-Won Lee

**Affiliations:** 1 Division of Rheumatology, Department of Internal Medicine, International St. Mary’s Hospital, Catholic Kwandong University College of Medicine, Incheon, Republic of Korea; 2 Division of Gastroenterology, Department of Internal Medicine, Yonsei University College of Medicine, Seoul, Republic of Korea; 3 Yonsei Liver Center, Severance Hospital, Seoul, Republic of Korea; 4 Division of Rheumatology, Department of Internal Medicine, Yonsei University College of Medicine, 50-1 Yonsei-ro, Seodaemun–gu, Seoul 03722 Republic of Korea; 5 Institute for Immunology and Immunological Diseases, Yonsei University College of Medicine, 50-1 Yonsei-ro, Seodaemun–gu, Seoul 03722 Republic of Korea

**Keywords:** Microscopic polyangiitis, Granulomatosis with polyangiitis, FIB-4, Mortality

## Abstract

**Background:**

The fibrosis-4 index (FIB-4) has been reported to be associated with all-cause mortality in several chronic diseases. In this study, we investigated whether at diagnosis could be associated with all-cause mortality in patients with microscopic polyangiitis (MPA) and granulomatosis with polyangiitis (GPA).

**Methods:**

We retrospectively reviewed the medical records of 132 MPA and GPA patients without chronic liver diseases. Conventional risk factors included old age (≥ 65 years), male gender, diabetes mellitus (DM) and hypertension (HTN) at diagnosis, and disease-related risk factor included GPA, antineutrophil cytoplasmic antibody, Birmingham vasculitis activity score (BVAS) and five factor score (FFS (2009)). The cut-off of FIB-4 for significant liver fibrosis (S2–4) was set at 1.45.

**Results:**

The mean age was 57.2 years and 27 patients (20.5%) had significant liver fibrosis (FIB-4 ≥ 1.45). Fifteen patients (11.4%) died during follow-up. In the univariable Cox Hazards model, age ≥ 65 years (Hazard ratio (HR) 5.055), DM (HR 3.446), HTN (HR 4.611), FFS (2009) ≥ 2 (HR 4.849) and FIB-4 ≥ 1.45 (HR 9.958) at diagnosis were significantly associated with all-cause mortality. In the multivariable Cox Hazards model, only FIB-4 at diagnosis ≥1.45 (HR 6.253, 95% confidence interval 1.398, 27.963) was associated with all-cause mortality during the follow-up in patients with MPA and GPA.

**Conclusions:**

FIB-4 at diagnosis ≥1.45 is an independent predictor of all-cause mortality during follow-up in patients with MPA and GPA, and furthermore its predictive potential is higher than those of conventional and AAV-related risk factors for all-cause mortality.

**Electronic supplementary material:**

The online version of this article (10.1186/s12876-019-1007-z) contains supplementary material, which is available to authorized users.

## Background

Antineutrophil cytoplasmic antibody (ANCA)-associated vasculitis (AAV) is a group systemic vasculitides, which can affect small vessels ranging from capillaries to intraparenchymal arterioles and venules of almost all the organs [1]. AAV consists of three variants, such as microscopic polyangiitis (MPA), granulomatosis with polyangiitis (GPA) and eosinophilic granulomatosis with polyangiitis (EGPA) [1, 2]. MPA and GPA mainly present similar pulmonary and renal symptoms despite different genetic and antigenic backgrounds, whereas EGPA often exhibit both allergic and necrotising vasculitic features [1–3].

Since AAV can bring out an amount of inflammatory burden and damage in major organs including lungs, kidneys and heart, AAV can be occasionally fatal. So far, there have been diverse reports regarding all-cause mortality in patients with AAV from different ethnic and geographic backgrounds: one year-cumulative patient survival rates were from 82 to 95% in Western countries [4], and that was reported to be 79.1% in Japan [5]. In Korean patients with AAV, 10-year cumulative patient survival rate was estimated up to 92.8% [6].

Renal and pulmonary involvements and five factor score (FFS (2009)) ≥ 2 at diagnosis of AAV have been reported as AAV-related risk factors for all-cause mortality [6, 7], together with conventional risk factors for all-cause mortality among the general population including age, male gender, diabetes mellitus (DM) and hypertension (HTN) [8]. Although infectious or immunosuppressive drug-related causes are major aetiologies of death in AAV patients, identifying predictors of all-cause mortality at diagnosis in immunosuppressive drug-naïve patients may have clinical implications in the real settings.

The fibrosis-4 index (FIB-4), which is calculated based on age, aspartate aminotransferase (AST), alanine aminotransferase (ALT) and platelet count, was first proposed to assess liver fibrosis in hepatitis C virus (HCV)-monoinfected patients [9]. The critical cut-off of FIB-4 for predicting significant liver fibrosis (S2 or greater) is currently set as 1.45 [10]. Recently, it has been reported that the baseline FIB-4 is significantly associated with poor outcomes in patients with liver diseases, such as hepatocellular carcinoma development or all-cause mortality [11, 12]. FIB-4 was also reported to be an independent risk factor of chronic kidney disease in patients with non-alcoholic fatty liver disease [13]. The association of FIB-4 with all-cause mortality was also demonstrated in patients with heart failure, which is a non-liver disease [14]. With this report, we searched previous reports on the association between the baseline FIB-4 and all-cause mortality in patients with AAV. However, to our best knowledge, there was no report regarding the association of FIB-4 at the time of diagnosis of AAV with all-cause mortality during follow-up to date. In our retrospective AAV-cohort, all-cause mortality was observed only in patients with MPA and GPA, but not EGPA [6]. Hence, in this study, we investigated whether FIB-4 at diagnosis could be associated with all-cause mortality in 132 immunosuppressive drug-naïve patients with MPA and GPA.

## Methods

### Patients

We retrospectively reviewed the medical records of 138 immunosuppressive drug-naïve patients with MPA and GPA based on the following inclusion criteria: i) patients who had been first classified as AAV at the Department of Internal Medicine, Yonsei University College of Medicine, Severance Hospital, from October 2000 to December 2017; ii) patients who fulfilled the American College of Rheumatology 1990 criteria and were reclassified by the 2007 European Medicines Agency algorithm, in which authors added the modified contents of the Chapel Hill Consensus Conferences (CHCC) Nomenclature of Vasculitis proposed in 2012 [1, 2]; iii) patients who had well-documented medical records with which to assess both clinical manifestations at diagnosis and death during follow-up, and calculate Birmingham vasculitis activity score (BVAS) and FFS (2009) at diagnosis [15, 16]. Because BVAS for GPA has a different weight-system compared to BVAS, we evenly applied BVAS to GPA to unify the scoring system; iv) patients who had results on perinuclear (P)-ANCA and cytoplasmic (C)-ANCA or myeloperoxidase (MPO)-ANCA and proteinase 3 (PR3)-ANCA levels at diagnosis. v) patients who had been followed up for 12 weeks or greater; vi) patients who had no medical history to affect either BVAS, ANCA positivity or FIB-4 prior to or at diagnosis, particularly, chronic liver diseases including viral hepatitis, coexisting malignancies, serious comorbidities and serious infection, which were identified in the 10th revised International Classification Diseases; vii) patients who had never received immunosuppressive drugs for AAV prior to diagnosis, which were searched by the Korean Drug Utilisation Review system. Of 138 AAV patients, 2 patients were excluded due to HBsAg positive and one patient was excluded due to anti-HCV positive. And furthermore, 2 patients and one patient were excluded due to alcoholic liver disease and non-alcoholic liver disease, respectively. Finally, we included 132 immunosuppressive drug-naïve patients with MPA (*N* = 91) and GPA (*N* = 41).

### Clinical and laboratory data

We obtained age at diagnosis and gender as demographic data and collected laboratory results including ANCA at diagnosis as described in Table 1. BVAS and FFS (2009) at diagnosis were calculated by reviewing the medical records. Comorbidities belonging to BVAS or FFS (2009), such as interstitial lung disease, diffuse alveolar haemorrhage, gastrointestinal bleeding, cardiovascular diseases, chronic kidney disease ≥ stage 3 and cerebrovascular accident, were excluded in AAV-related risk factors for all-cause mortality. In this study, we assessed old age (≥ 65 years), male gender, DM and HTN at diagnosis as conventional risk factors, and GPA, ANCA positivity at diagnosis, BVAS at diagnosis ≥16 and FFS (2009) at diagnosis ≥2 as AAV-related risk factors for all-cause mortality during follow-up according to the previous studies [6, 8]. We also evaluate the predictive potential of FIB-4 at diagnosis ≥1.45 for all-cause mortality for the follow-up period. We defined the follow-up duration as the period from diagnosis to the last visit in survived patients, whereas we defined it as the period from diagnosis to death in deceased patients.

**Table 1:** Baseline characteristics of patients with MPA and GPA (*N* = 132)

Variables	Values
Demographic data	
Age at diagnosis (year old)	57.2 ± 14.7
Male gender (N, (%))	40 (30.3)
AAV variants (N, (%))	
MPA	91 (68.9)
GPA	41 (31.1)
ANCA positivity (N, (%))	112 (84.8)
Activity and prognostic factor at diagnosis	
BVAS	13.1 ± 7.2
FFS (2009)	1.4 ± 1.0
FFS (2009) ≥ 2 (N, (%))	57 (43.2)
Laboratory results at diagnosis	
White blood cell (/mm^3^)	9538.3 ± 3940.5
Haemoglobin (g/dL)	12.0 ± 1.2
Platelet × 10^3^ (/mm^3^)	331.8 ± 144.7
Prothrombin time (INR)	1.0 ± 0.1
Fasting glucose (mg/dL)	116.8 ± 44.6
Blood urea nitrogen (mg/dL)	28.1 ± 25.3
Creatinine (mg/dL)	1.9 ± 2.1
Protein (g/dL)	6.7 ± 0.9
Serum albumin (g/dL)	3.5 ± 0.8
Alkaline phosphatase (IU/L)	93.7 ± 94.3
Aspartate aminotransferase (IU/L)	23.2 ± 26.5
Alanine aminotransferase (IU/L)	23.4 ± 39.3
Total bilirubin (mg/dL)	0.7 ± 1.6
Total cholesterol (mg/dL)	171.4 ± 50.4
Acute reactants at diagnosis	
Erythrocyte sedimentation rate (mm/hr)	62.8 ± 38.0
C-reactive protein (mg/L)	44.0 ± 56.8
Liver fibrosis index at diagnosis	
FIB-4	1.1 ± 0.9
FIB-4 ≥ 1.45 (N, (%))	27 (20.5)
Comorbidities except items of BVAS or FFS (2009) at diagnosis^a^ (N, (%))	
DM	26 (19.7)
HTN	61 (46.2)
Follow-up duration (months)	52.4 ± 52.2
Death (N, (%))	15 (11.4%)

Values are expressed as a mean ± standard deviation and number (N) (%)

^a^ interstitial lung disease, diffuse alveolar haemorrhage, gastrointestinal bleeding, cardiovascular diseases, chronic kidney disease ≥ stage 3 and cerebrovascular accident and so on

*AAV* ANCA-associated vasculitis, *ANCA* antineutrophil cytoplasmic antibody, *MPA* microscopic polyangiitis, *GPA* granulomatosis with polyangiitis, *BVAS* Birmingham vasculitis activity score, *FFS* five factor score, *FIB-4* fibrosis-4, *DM* diabetes mellitus, *HTN* hypertension

### Equations of FIB-4 and significant liver fibrosis

FIB-4 = age (years) x AST (IU/L) / platelet count (10^9^/L)/√ALT (IU/L) [9]. The critical cut-off of FIB-4 for significant liver fibrosis (S2–4) was set at 1.45 [10].

### Statistical analyses

All statistical analyses were conducted using SPSS software (version 23 for windows; IBM Corp., Armonk, NY, USA). Continuous variables were expressed as a mean ± standard deviation, and categorical variables were expressed as a number (percentage). The multivariable Cox hazard model using variables with statistical significance in the univariable Cox hazard model was conducted to appropriately obtain the hazard ratios (HRs) during the considerable follow-up duration. We stratified AAV patients into three groups based on the tertile of BVAS and defined the lower limit of the highest tertile as the cut-off for the current severe AAV (BVAS at diagnosis ≥16). The odds ratio (OR) was assessed using the multivariable logistic regression analysis of variables with *p*-values less than 0.05 in the univariable logistic regression analysis. *P*-values less than 0.05 were considered statistically significant.

## Results

### Baseline characteristics of 132 patients with MPA and GPA

The baseline characteristics were described in Table 1. The mean age at diagnosis was 57.2 years and 40 patients (30.3%) were men. Ninety-one patients (68.9) were classified as MPA and 41 patients (31.1%) were as GPA. Any type of ANCA was detected in 112 patients (84.8%). The mean BVAS and FFS (2009) at diagnosis were 13.1 and 1.4 and 57 patients (43.2%) had FFS (2009) at diagnosis ≥2. The mean levels of AST and ALT at diagnosis were 23.2 IU/L and 23.4 IU/L, and the mean platelet count at diagnosis was 331,800.0/mm^3^. The mean FIB-4 at diagnosis was 1.1 and 27 patients (20.5%) had significant liver fibrosis (FIB-4 ≥ 1.45). At diagnosis, 26 patients (19.7%) had DM and 61 patients (46.2%) had HTN. The mean follow-up duration was 52.4 months and 15 patients (11.4%) died. During follow-up, glucocorticoid was the most frequently administered drug (84.8%), followed by cyclophosphamide (43.2%) and azathioprine (24.2%).

### Univariable cox hazards model analysis of risk factors for all-cause mortality

Among conventional risk factors for all-cause mortality, age at diagnosis ≥65 years (HR 5.055, 95% confidence interval 1.593, 16.039), DM at diagnosis (HR 3.446, 95% CI 1.111, 10.692) and HTN at diagnosis (HR 4.611, 95% CI 1.016, 20.927) were significantly associated with all-cause mortality Among AAV-related risk factor for all-cause mortality, only FFS (2009) at diagnosis ≥2 (HR 4.849, 95% CI 1.341, 17.537) was significantly associated with all-cause mortality. In addition, FIB-4 at diagnosis ≥1.45 (HR 9.958, 95% CI 2.550, 38.877) also exhibited a significant association with all-cause mortality during follow-up in patients with MPA and GPA (Table 2).

**Table 2:** Univariable Cox Hazards model analysis of conventional and AAV-related risk factors for all-cause mortality in patients with MPA and GPA

Variables	HR	95% confidence interval	*P*-value
Age at diagnosis ≥65 years	5.055	1.593, 16.039	0.006
Male gender	0.557	0.195, 1.587	0.273
DM at diagnosis	3.446	1.111, 10.692	0.032
HTN at diagnosis	4.611	1.016, 20.927	0.048
GPA versus MPA	1.002	0.311, 3.226	0.997
ANCA positivity at diagnosis	3.423	0759, 15.438	0.109
BVAS at diagnosis ≥16	1.554	0.553, 4.370	0.403
FFS (2009) at diagnosis ≥2	4.849	1.341, 17.537	0.016
FIB-4 at diagnosis ≥1.45	9.958	2.550, 38.877	0.001

*AAV* ANCA-associated vasculitis, *ANCA* antineutrophil cytoplasmic antibody, *GPA* granulomatosis with polyangiitis, *MPA* microscopic polyangiitis, *BVAS* Birmingham vasculitis activity score, *FFS* five factor score, *FIB-4* fibrosis-4, *DM* diabetes mellitus, *HTN* hypertension

### Multivariable cox hazards model analysis of risk factors for all-cause mortality

We also conducted the multivariable Cox hazards model using variables with statistical significance in the univariable Cox hazards model. Among age ≥ 65 years, DM, HTN, FFS (2009) ≥ 2 and FIB-4 ≥ 1.45 at diagnosis, only FIB-4 at diagnosis ≥1.45 was an independent predictor of all-cause mortality during the follow-up in patients with MPA and GPA (Table 3).

**Table 3:** Multivariable Cox Hazards model analysis of conventional and AAV-related risk factors for all-cause mortality in patients with MPA and GPA

Variables	Including age at diagnosis ≥ 65 years		
	HR	95% confidence interval	*P*-value
Age at diagnosis ≥65 years	2.491	0.656, 9.459	0.180
DM at diagnosis	1.581	0.426, 5.864	0.493
HTN at diagnosis	1.727	0.332, 8.987	0.516
FFS (2009) at diagnosis ≥2	2.921	0.705, 12.095	0.139
FIB-4 at diagnosis ≥1.45	6.253	1.398, 27.963	0.016

*AAV* ANCA-associated vasculitis, *ANCA* antineutrophil cytoplasmic antibody, *GPA* granulomatosis with polyangiitis, *MPA* microscopic polyangiitis, *BVAS* Birmingham vasculitis activity score, *FFS* five factor score, *FIB-4* fibrosis-4, *DM* diabetes mellitus, *HTN* hypertension

## Discussion

In this study, we compared the predictive potential of FIB-4 at diagnosis ≥1.45 with those of conventional and AAV-related risk factors for all-cause mortality in 132 immunosuppressive drug-naïve patients with MPA and GPA. In the multivariable Cox Hazard model analysis, old age (≥ 65 years), DM, HTN, FFS (2009) ≥ 2 and FIB-4 ≥ 1.45 at diagnosis were included. Interestingly, we found that only FIB-4 at diagnosis ≥1.45 was associated with all-cause mortality during follow-up in patients MPA and GPA. We considered two reasons why FIB-4 ≥ 1.45 at diagnosis exhibited the relatively high predictive power for all-cause mortality. First, inflammation may accelerate systemic fibrotic change through various inflammatory signals. Thus, the extent of liver fibrosis may indirectly reflect the accumulated amount of inflammatory burden in non-liver diseases such as heart failure [14]. We assume that FIB-4 at diagnosis could predict all-cause mortality in MPA and GPA patients in a similar manner. Next, we assume that FIB-4 might be influenced by both conventional and AAV-related risk factors for all-cause mortality prior to or at the time of diagnosis. To prove this assumption, we conducted the univariable and multivariable logistic regression analysis based on FIB-4 at diagnosis ≥1.45 using those risk factors at diagnosis. In the univariable analysis, age at diagnosis ≥65 years (OR 3.812) and DM at diagnosis (OR 5.200) were significantly associated with FIB-4 at diagnosis ≥1.45. BVAS at diagnosis ≥16 and FFS (2009) at diagnosis ≥2 exhibited a tendency to be associated with FIB-4, so they were also included in the multivariable analysis. In the multivariable analysis, only age at diagnosis ≥65 years (OR 3.088, 95% CI 1.157, 8.239) and DM at diagnosis (OR 4.556, 95% CI 1.678, 12.368) were significantly associated with FIB-4 at diagnosis ≥1.45 (Additional file 1: Table S1).

Since, age is one of variables comprising an equation of FIB-4, it is naturally accepted that FIB-4 may be directly correlated with age [9]. Whereas, pre-existing DM could induce non-alcoholic fatty liver disease, one of the systemic complication of DM, which can lead to an increase in FIB-4 within 3 years from baseline [17, 18]. The link between male gender and FIB-4 is controversial: according to a previous review regarding an effect of gender on the outcome of liver diseases, differences in incidence and severity of liver diseases between men and women may vary based on a disease-type [19]. The relation between pre-existing systemic HTN and FIB-4 is also controversial: non-alcoholic fatty liver disease was identified as an independent risk factor for the development of systemic HTN [20]. Therefore, we conclude that FIB-4 at diagnosis itself may be an independent predictor of all-cause mortality in MPA and GPA patients and at the same time, it may reflect the effect of old age and DM on subclinical liver fibrosis, leading to an increase in FIB-4 at diagnosis.

Despite no significant association in the multivariable logistic regression analysis, BVAS and FFS (2009) at diagnosis exhibited a tendency to be associated with FIB-4 at diagnosis. In cases of severe MPA and GPA, as a counterpart of TH1 and TH17 cells, which are important participants in the pathogenesis of AAV, Treg cells may enhance the production of transforming growth factors (TGF)-β, which can initiate and accelerate liver fibrosis [21]. Moreover, the higher extent of inflammatory burden may provoke the augmented production of reactive oxygen species, which can subsequently promote cell differentiation of fibroblasts to myoblasts in liver [22]. Since BVAS and FFS (2009) were calculated at diagnosis of MPA and GPA, together with FIB-4 at diagnosis, the effect of BVAS and FFS (2009) at diagnosis on FIB-4 at diagnosis might be negligible. However, considering an asymptomatic latency prior to diagnosis of MPA and GPA, BVAS and FFS (2009) at diagnosis might theoretically influence FIB-4 at diagnosis. Therefore, we also conclude that FIB-4 at diagnosis may reflect the subtle effect of the inflammatory burden of AAV on subclinical liver fibrosis, leading to an increase in FIB-4 at diagnosis.

In addition to conventional and AAV-related risk factors, we investigated the predictive potential of comorbidities at diagnosis for all-cause mortality. Interstitial lung disease at diagnosis (HR 6.981, 95% CI 1.535, 31.756) was significantly associated with all-cause mortality in MPA and GPA patients. However, chronic kidney disease over stage 3 (HR 2.615, 95% CI 0.883, 7.742), ischaemic heart disease (HR 3.493, 95% 0.703, 17.361) and cerebrovascular disease (HR 1.213, 95% CI 0.324, 4.546) at diagnosis exhibited no significant association with all-cause mortality. We added interstitial lung disease at diagnosis to the multivariable Cox Hazards model analysis together with 5 variables described in Table 3. Nevertheless, only FIB-4 at diagnosis ≥1.45 was significantly associated with all-cause mortality. Because interstitial lung disease is not an established conventional or AAV-related risk factor for all-cause mortality, we did not include interstitial lung disease in the multivariable Cox Hazards model in this study.

In this study, we first demonstrated that FIB-4 at diagnosis ≥1.45 is an independent predictor of all-cause mortality during follow-up in patients with MPA and GPA, and furthermore its predictive potential is higher than those of conventional and AAV-related risk factors for all-cause mortality. However, our study also has several issues. First, despite the significant association between FIB-4 at diagnosis and all-cause mortality, we could not clarify the direct mechanism of FIB-4 at diagnosis to estimate all-cause mortality. Second, we could not provide the concrete data on liver fibrosis by liver histology or transient elastography. Third, our study was designed as a retrospective study, we could not strictly control the confounding factors. Particularly, we could not confirm the repeated results of ANCAs in patients without ANCA. Fourth, this study was conducted in a single centre, the number of deceased patients was too small to augment the statistical power. Future prospective and multi-centric studies with a larger number of patients will validate the clinical significance of FIB-4 at diagnosis in predicting all-cause mortality during follow-up of MPA and GPA in real-world clinical practice.

## Conclusions

FIB-4 at diagnosis ≥1.45 is an independent predictor of all-cause mortality during follow-up in patients with MPA and GPA, and furthermore its predictive potential is higher than those of conventional and AAV-related risk factors for all-cause mortality.

## Additional file

Additional file 1: **Table S1.** Univariable and multivariable logistic regression of conventional and AAV-related risk factors for FIB-4 at diagnosis ≥ 1.45 in patients with MPA and GPA. (DOCX 20 kb)

## Data Availability

The data used and analysed in this study are available from the corresponding author on reasonable request.

## Abbreviations

AAV: ANCA-associated vasculitis
ALT: Alanine aminotransferase
ANCA: Antineutrophil cytoplasmic antibody
AST: Aspartate aminotransferase
BVAS: Birmingham vasculitis activity score
C: Cytoplasmic
CHCC: The Chapel Hill Consensus Conferences
DM: Diabetes mellitus
EGPA: Eosinophilic granulomatosis with polyangiitis
FFS: Five factor score
FIB-4: Fibrosis-4
GPA: Granulomatosis with polyangiitis
HCV: Hepatitis C virus
HTN: Hypertension
MPA: Microscopic polyangiitis
MPO: Myeloperoxidase
P: Perinuclear
PR3: Proteinase 3
TGF: Transforming growth factors
