# Supplementary material for: Fibrosis-4 index at diagnosis is associated with all-cause mortality in patients with microscopic polyangiitis and granulomatosis with polyangiitis
Source: BMC Gastroenterol. 2019 Jun 13;19:90. doi: 10.1186/s12876-019-1007-z (PMC6567497; doi:10.1186/s12876-019-1007-z)
Supplement: Supplementary file 1 — Table S1. Univariable and multivariable logistic regression of conventional and AAV-related risk factors for FIB-4 at diagnosis ≥ 1.45 in patients with MPA and GPA. (DOCX 20 kb) [file 12876_2019_1007_MOESM1_ESM.docx]

**Supplementary Table 1 Univariable and multivariable logistic regression of conventional and AAV-related risk factors for FIB-4 at diagnosis ≥ 1.45 in patients with MPA and GPA**

| **Variables** | **Univariable** | | | **Multivariable** | | |
| --- | --- | --- | --- | --- | --- | --- |
|  | **OR** | **95% confidence interval** | **P-value** | **OR** | **95% confidence interval** | **P-value** |
| Age at diagnosis ≥ 65 years | 3.812 | 1.583, 9.179 | 0.003 | 3.088 | 1.157, 8.239 | 0.024 |
| Male gender | 0.838 | 0.339, 2.068 | 0.701 |  |  |  |
| DM at diagnosis | 5.200 | 2.021, 13.377 | 0.001 | 4.556 | 1.678, 12.368 | 0.003 |
| HTN at diagnosis | 1.603 | 0.684, 3.756 | 0.277 |  |  |  |
| GPA versus MPA | 0.731 | 0.282, 1.895 | 0.519 |  |  |  |
| ANCA positivity at diagnosis | 0.733 | 0.241, 2.235 | 0.585 |  |  |  |
| BVAS at diagnosis ≥ 16 | 2.249 | 0.953, 5.306 | 0.064 | 1.963 | 0.719, 2.856 | 0.188 |
| FFS (2009) at diagnosis ≥ 2 | 2.271 | 0.959, 5.376 | 0.062 | 0.986 | 0.340, 2.856 | 0.979 |

AAV: ANCA-associated vasculitis; ANCA: antineutrophil cytoplasmic antibody; GPA: granulomatosis with polyangiitis; MPA: microscopic polyangiitis; BVAS: Birmingham vasculitis activity score; FFS: five factor score; FIB-4: fibrosis-4; DM: diabetes mellitus; HTN: hypertension.
